# Structural Brain Changes in Chronic Pain Reflect Probably Neither Damage Nor Atrophy

**DOI:** 10.1371/journal.pone.0054475

**Published:** 2013-02-06

**Authors:** Rea Rodriguez-Raecke, Andreas Niemeier, Kristin Ihle, Wolfgang Ruether, Arne May

**Affiliations:** 1 Department of Systems Neuroscience, University Medical Center Hamburg Eppendorf, Hamburg, Germany; 2 Department of Orthopaedics, University Medical Center Hamburg Eppendorf, Hamburg, Germany; University of Regensburg, Germany

## Abstract

Chronic pain appears to be associated with brain gray matter reduction in areas ascribable to the transmission of pain. The morphological processes underlying these structural changes, probably following functional reorganisation and central plasticity in the brain, remain unclear. The pain in hip osteoarthritis is one of the few chronic pain syndromes which are principally curable. We investigated 20 patients with chronic pain due to unilateral coxarthrosis (mean age 63.25±9.46 (SD) years, 10 female) before hip joint endoprosthetic surgery (pain state) and monitored brain structural changes up to 1 year after surgery: 6–8 weeks, 12–18 weeks and 10–14 month when completely pain free. Patients with chronic pain due to unilateral coxarthrosis had significantly less gray matter compared to controls in the anterior cingulate cortex (ACC), insular cortex and operculum, dorsolateral prefrontal cortex (DLPFC) and orbitofrontal cortex. These regions function as multi-integrative structures during the experience and the anticipation of pain. When the patients were pain free after recovery from endoprosthetic surgery, a gray matter increase in nearly the same areas was found. We also found a progressive increase of brain gray matter in the premotor cortex and the supplementary motor area (SMA). We conclude that gray matter abnormalities in chronic pain are not the cause, but secondary to the disease and are at least in part due to changes in motor function and bodily integration.

## Introduction

Evidence of functional and structural reorganization in chronic pain patients support the idea that chronic pain should not only be conceptualized as an altered functional state, but also as a consequence of functional and structural brain plasticity [1], [2], [3], [4], [5], [6]. In the last six years, more than 20 studies were published demonstrating structural brain changes in 14 chronic pain syndromes. A striking feature of all of these studies is the fact that the gray matter changes were not randomly distributed, but occur in defined and functionally highly specific brain areas – namely, involvement in supraspinal nociceptive processing. The most prominent findings were different for each pain syndrome, but overlapped in the cingulate cortex, the orbitofrontal cortex, the insula and dorsal pons [4]. Further structures comprise the thalamus, dorsolateral prefrontal cortex, basal ganglia and hippocampal area. These findings are often discussed as cellular atrophy, reinforcing the idea of damage or loss of brain gray matter [7], [8], [9]. In fact, researchers found a correlation between brain gray matter decreases and duration of pain [6], [10]. But the duration of pain is also linked to the patient’s age, and the age dependent global, but also regionally specific decline of gray matter is well documented [11]. On the other hand, these structural changes could also be a decrease in cell size, extracellular fluids, synaptogenesis, angiogenesis or even due to blood volume changes [4], [12], [13]. Whatever the source is, for our interpretation of such findings it is important to see these morphometric findings in the light of a wealth of morphometric studies in exercise dependant plasticity, given that regionally specific structural brain changes have been repeatedly shown following cognitive and physical exercise [14].

It is not understood why only a relatively small proportion of humans develop a chronic pain syndrome, considering that pain is a universal experience. The question arises whether in some humans a structural difference in central pain transmitting systems may act as a diathesis for chronic pain. Gray matter changes in phantom pain due to amputation [15] and spinal cord injury [3] indicate that the morphological changes of the brain are, at least in part, a consequence of chronic pain. However, the pain in hip osteoarthritis (OA) is one of the few chronic pain syndrome which is principally curable, as 88% of these patients are regularly free of pain following total hip replacement (THR) surgery [16]. In a pilot study we have analysed ten patients with hip OA before and shortly after surgery. We found decreases of gray matter in the anterior cingulated cortex (ACC) and insula during chronic pain before THR surgery and found increases of gray matter in the corresponding brain areas in the pain free condition after surgery [17]. Focussing on this result, we now expanded our studies investigating more patients (n = 20) after successful THR and monitored structural brain changes in four time intervals, up to one year following surgery. To control for gray matter changes due to motor improvement or depression we also administered questionnaires targeting improvement of motor function and mental health.

## Materials and Methods

### Volunteers

The patients reported here are a subgroup of 20 patients out of 32 patients published recently who were compared to an age- and gender-matched healthy control group [17] but participated in an additional one year follow-up investigation. After surgery 12 patients dropped out because of a second endoprosthetic surgery (n = 2), severe illness (n = 2) and withdrawal of consent (n = 8). This left a group of twenty patients with unilateral primary hip OA (mean age 63.25±9.46 (SD) years, 10 female) who were investigated four times: before surgery (pain state) and again 6–8 and 12–18 weeks and 10–14 months after endoprosthetic surgery, when completely pain free. All patients with primary hip OA had a pain history longer than 12 months, ranging from 1 to 33 years (mean 7.35 years) and a mean pain score of 65.5 (ranging from 40 to 90) on a visual analogue scale (VAS) ranging from 0 (no pain) to 100 (worst imaginable pain). We assessed any occurrence of minor pain events, including tooth-, ear- and headache up to 4 weeks prior to the study. We also randomly selected the data from 20 sex- and age matched healthy controls (mean age 60,95±8,52 (SD) years, 10 female) of the 32 of the above mentioned pilot study [17]. None of the 20 patients or of the 20 sex- and age matched healthy volunteers had any neurological or internal medical history. The study was given ethical approval by the local Ethics committee and written informed consent was obtained from all study participants prior to examination.

### Behavioural Data

We collected data on depression, somatization, anxiety, pain and physical and mental health in all patients and all four time points using the following standardized questionnaires: Beck Depression Inventory (BDI) [18], Brief Symptom Inventory (BSI) [19], Schmerzempfindungs-Skala (SES = pain unpleasantness scale) [20] and Health Survey 36-Item Short Form (SF-36) [21] and the Nottingham Health Profile (NHP). We conducted repeated measures ANOVA and paired two-tailed t-Tests to analyse the longitudinal behavioural data using SPSS 13.0 for Windows (SPSS Inc., Chicago, IL), and used Greenhouse Geisser correction if the assumption for sphericity was violated. The significance level was set at p<0.05.

### VBM - Data Acquisition

#### Image acquisition

High-resolution MR scanning was performed on a 3T MRI system (Siemens Trio) with a standard 12-channel head coil. For each of the four time points, scan I (between 1 day and 3 month before endoprosthetic surgery), scan II (6 to 8 weeks after surgery), scan III (12 to 18 weeks after surgery) and scan IV (10–14 months after surgery), a T1 weighted structural MRI was acquired for each patient using a 3D-FLASH sequence (TR 15 ms, TE 4.9 ms, flip angle 25°, 1 mm slices, FOV 256×256, *voxel size 1*×*1*×*1 mm*).

### Image Processing and Statistical Analysis

Data pre-processing and analysis were performed with SPM2 (Wellcome Department of Cognitive Neurology, London, UK) running under Matlab (Mathworks, Sherborn, MA, USA) and containing a voxel-based morphometry (VBM)-toolbox for longitudinal data, that is based on high resolution structural 3D MR images and allows for applying voxel-wise statistics to detect regional differences in gray matter density or volumes [22], [23]. In summary, pre-processing involved spatial normalization, gray matter segmentation and 10 mm spatial smoothing with a Gaussian kernel. For the pre-processing steps, we used an optimized protocol [22], [23] and a scanner- and study-specific gray matter template [17]. We used SPM2 rather than SPM5 or SPM8 to make this analysis comparable to our pilot study [17]. as it allows an excellent normalisation and segmentation of longitudinal data. However, as a more recent update of VBM (VBM8) became available recently (http://dbm.neuro.uni-jena.de/vbm/), we also used VBM8.

### Cross-sectional Analysis

We used a two-sample t-test in order to detect regional differences in brain gray matter between groups (patients at time point scan I (chronic pain) and healthy controls). We applied a threshold of p<0.001 (uncorrected) across the whole brain because of our strong *a priory* hypothesis, which is based on 9 independent studies and cohorts showing decreases in gray matter in chronic pain patients [7], [8], [9], [15], [24], [25], [26], [27], [28], that gray matter increases will appear in the same (for pain processing relevant) regions as in our pilot study (17). The groups were matched for age and sex with no significant differences between the groups. To investigate whether the differences between groups changed after one year, we also compared patients at time point scan IV (pain free, one year follow-up) to our healthy control group.

### Longitudinal Analysis

To detect differences between time points (Scan I–IV) we compared the scans before surgery (pain state) and again 6–8 and 12–18 weeks and 10–14 months after endoprosthetic surgery (pain free) as repeated measure ANOVA. Because any brain changes due to chronic pain may need some time to recede following operation and cessation of pain and because of the post surgery pain the patients reported, we compared in the longitudinal analysis scan I and II with scan III and IV. For detecting changes that are not closely linked to pain, we also looked for progressive changes over all time intervals. We flipped the brains of patients with OA of the left hip (n = 7) in order to normalize for the side of the pain for both, the group comparison and the longitudinal analysis, but primarily analysed the unflipped data. We used the BDI score as a covariate in the model.

## Results

### Behavioral Data

All patients reported chronic hip pain before surgery and were pain free (regarding this chronic pain) immediately after surgery, but reported rather acute post-surgery pain on scan II which was different from the pain due to osteoarthritis. The mental health score of the SF-36 (F(1.925/17.322) = 0.352, p = 0.7) and the BSI global score GSI (F(1.706/27.302) = 3.189, p = 0.064) showed no changes over the time course and no mental co-morbidity. None of the controls reported any acute or chronic pain and none showed any symptoms of depression or physical/mental disability.

Before surgery, some patients showed mild to moderate depressive symptoms in BDI scores that significantly decreased on scan III (t(17) = 2.317, p = 0.033) and IV (t(16) = 2.132, p = 0.049). Additionally, the SES scores (pain unpleasantness) of all patients improved significantly from scan I (before the surgery) to scan II (t(16) = 4.676, p<0.001), scan III (t(14) = 4.760, p<0.001) and scan IV (t(14) = 4.981, p<0.001, 1 year after surgery) as pain unpleasantness decreased with pain intensity. The pain rating on scan 1 and 2 were positive, the same rating on day 3 and 4 negative. The SES only describes the quality of perceived pain. It was therefore positive on day 1 and 2 (mean 19.6 on day 1 and 13.5 on day 2) and negative (n.a.) on day 3 & 4. However, some patients did not understand this procedure and used the SES as a global “quality of life” measure. This is why all patients were asked on the same day individually and by the same person regarding pain occurrence.

In the short form health survey (SF-36), which consists of the summary measures of a *Physical Health Score* and a *Mental Health Score* [29], the patients improved significantly in *the Physical Health score* from scan I to scan II (t(17) = −4.266, p = 0.001), scan III (t(16) = −8.584, p<0.001) and IV (t(12) = −7.148, p<0.001), but not in the *Mental Health Score*. The results of the NHP were similar, in the subscale “pain” (reversed polarity) we observed a significant change from scan I to scan II (t(14) = −5.674, p<0.001, scan III (t(12) = −7.040, p<0.001 and scan IV (t(10) = −3.258, p = 0.009). We also found a significant increase in the subscale “physical mobility” from scan I to scan III (t(12) = −3.974, p = 0.002) and scan IV (t(10) = −2.511, p = 0.031). There was no significant change between scan I and scan II (six weeks after surgery).

### Structural Data

#### Cross-sectional analysis

**Figure 1:**
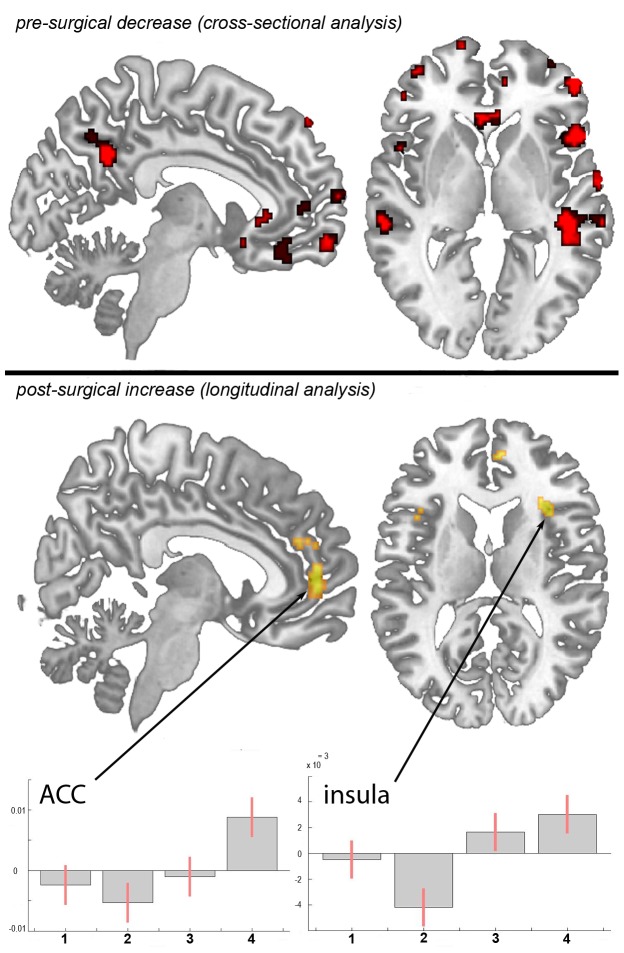
Statistical parametric maps demonstrating the structural differences in gray matter in patients with chronic pain due to primary hip OA compared to controls and longitudinally compared to themselves over time. Significant gray matter changes are shown superimposed in color, cross-sectional data is depicted in red and longitudinal data in yellow. Axial plane: the left side of the picture is the left side of the brain. top: Areas of significant decrease of gray matter between patients with chronic pain due to primary hip OA and unaffected control subjects. p<0.001 uncorrected bottom: Gray matter increase in 20 pain free patients at the third and fourth scanning period after total hip replacement surgery, as compared to the first (preoperative) and second (6–8 weeks post surgery) scan. p<0.001 uncorrected Plots: Contrast estimates and 90% Confidence interval, effects of interest, arbitrary units. x-axis: contrasts for the 4 timepoints, y-axis: contrast estimate at −3, 50, 2 for ACC and contrast estimate at 36, 39, 3 for insula.

We included age as a covariate in the general linear model and found no age confounds. Compared to sex and age matched controls, patients with primary hip OA (n = 20) showed pre-operatively (Scan I) reduced gray matter in the anterior cingulate cortex (ACC), the insular cortex, operculum, dorsolateral prefrontal cortex (DLPFC), right temporal pole and cerebellum (Table 1 and Figure 1). Except for the right putamen (x = 31, y = −14, z = −1; p<0.001, t = 3.32) no significant increase in gray matter density was found in patients with OA compared to healthy controls. Comparing patients at time point scan IV with matched controls, the same results were found as in the cross-sectional analysis using scan I compared to controls.

**Table 1:** Cross-sectional data (not flipped, patients with OA<healthy controls), gray matter decrease in OA (p<0.001 uncorrected).

	MNI				
Anatomical locations	x	y	z	Peak t-scores	number of voxels
anterior cingulate cortex	−12	25	−16	5.51	2678
insular cortex/operculum	54	22	5	5.00	1527
Cerebellum	26	−42	−27	4.16	1913
pars orbitalis/orbital gyrus	48	47	−9	3.77	1080
superior frontal gyrus	−14	68	2	3.97	96
mid temporal gyrus	−63	−29	−7	4.15	1961
superior medial gyrus	10	67	13	4.35	222
pars opercularis	48	18	0	4.25	1527
DLPFC	45	35	29	4.69	1332
superior temporal gyrus/S2	64	−5	4	4.37	324

Flipping the data of patients with left hip OA (n = 7) and comparing them with healthy controls did not change the results significantly, but for a decrease in the thalamus (x = 10, y = −20, z = 3, p<0.001, t = 3.44) and an increase in the right cerebellum (x = 25, y = −37, z = −50, p<0.001, t = 5.12) that did not reach significance in the unflipped data of the patients compared to controls.

#### Longitudinal analysis

**Figure 2:**
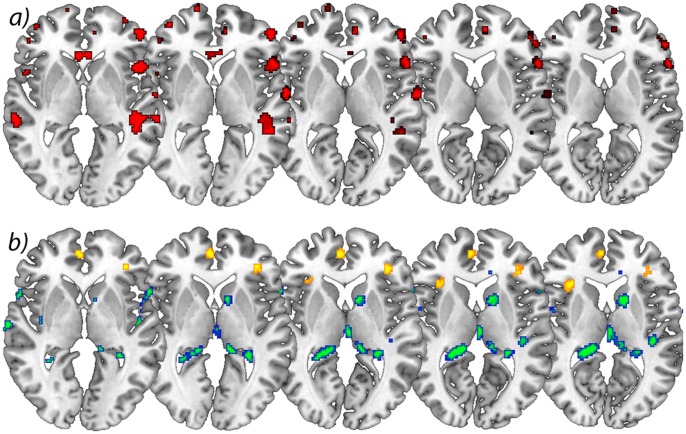
**a)** Significant increases in brain gray matter following successful operation. Axial view of significant decrease of gray matter in patients with chronic pain due to primary hip OA compared to control subjects. p<0.001 uncorrected (cross-sectional analysis), **b)** Longitudinal increase of gray matter over time in yellow comparing scan I&II<scan III<scan IV) and longitudinal decrease of gray matter over time in blue/green comparing scan I&II>scan III>scan IV) in patients with OA. p<0.001 uncorrected (longitudinal analysis). The left side of the picture is the left side of the brain.

In the longitudinal analysis, a significant increase (p<.001 uncorrected) of gray matter was detected by comparing the first and second scan (chronic pain/post-surgery pain) with the third and fourth scan (pain free) in the ACC, insular cortex, cerebellum and pars orbitalis in the patients with OA (Table 2 and Figure 1). Gray matter decreased over time (p<.001 whole brain analysis uncorrected) in the secondary somatosensory cortex, hippocampus, midcingulate cortex, thalamus and caudate nucleus in patients with OA (Figure 2).

**Table 2:** Longitudinal data, significant changes in gray matter in patients with chronic pain due to primary hip osteoarthritis.

	MNI					
Anatomical locations	x	y	z	Peak t-scores	number of voxels	Increase/decrease
anterior cingulate cortex	−3	50	2	4.21	674	increase
insular cortex	−33	21	13	4.13	420	increase
pars orbitalis	4	39	10	3.77	77	increase
Cerebellum	47	−71	−22	5.00	5139	increase
mid cingulate cortex	11	−26	43	5.04	1713	decrease
caudate nucleus	11	9	8	4.14	791	decrease
pars opercularis	−58	16	2	4.53	445	decrease
Area 44	64	9	8	4.59	1155	decrease
Thalamus	3	−17	6	4.17	1540	decrease
operculum/S2	−66	−26	17	5.66	2935	decrease
Operculum	41	−26	11	4.41	283	decrease
Hippocampus	−18	−37	7	4.50	1662	decrease

The changes are tabulated in terms of the brain region and the corresponding Brodmann’s area (BA). The x, y, z co-ordinates are according to the MNI atlas. Each location is the peak within a cluster (defined as the voxel with the highest T-score). (Patients with OA, not flipped) Increase = increase in gray matter scan I/scan II>scan III>scan IV. Decrease = decrease in gray matter scan I/scan II<scan III<scan IV; p<0.001 uncorrected).

Flipping the data of patients with left hip OA (n = 7) did not change the results significantly, but for a decrease of brain gray matter in the Heschl’s Gyrus (x = −41, y = −21, z = 10, p<0.001, t = 3.69) and Precuneus (x = 15, y = −36, z = 3, p<0.001, t = 4.60).

By contrasting the first scan (presurgery) with scans 3+4 (postsurgery), we found an increase of gray matter in the frontal cortex and motor cortex (p<0.001 uncorrected). We note that this contrast is less stringent as we have now less scans per condition (pain vs. non-pain). When we lower the threshold we repeat what we have found using contrast of 1+2 vs. 3+4.

By looking for areas that increase over all time intervals, we found changes of brain gray matter in motor areas (area 6) in patients with coxarthrosis following total hip replacement (scan I<scan II<scan III<scan IV)). Adding the BDI scores as a covariate did not change the results. Using the recently available software tool VBM8 including DARTEL normalisation (http://dbm.neuro.uni-jena.de/vbm/) we could replicate this finding in the anterior and mid-cingulate cortex and both anterior insulae.

We calculated the effect sizes and the cross-sectional analysis (patients vs. controls) yielded a Cohen’s d of 1.78751 in the peak voxel of the ACC (x = −12, y = 25, z = −16). We also calculated Cohen’s d for the longitudinal analysis (contrasting scan 1+2 vs. scan 3+4). This resulted in a Cohen’s d of 1.1158 in the ACC (x = −3, y = 50, z = 2). Regarding the insula (x = −33, y = 21, z = 13) and related to the same contrast, Cohen’s d is 1.0949. Additionally, we calculated the mean of the non-zero voxel values of the Cohen’s d map within the ROI (comprised of the anterior division of the cingulate gyrus and the subcallosal cortex, derived from the Harvard-Oxford Cortical Structural Atlas): 1.251223.

## Discussion

Monitoring whole brain structure over time, we confirm and expand our pilot data published recently [17]. We found changes in brain gray matter in patients with primary hip osteoarthritis in the chronic pain state, which reverse partly when these patients are pain free, following hip joint endoprosthetic surgery. The partial increase in gray matter after surgery is nearly in the same areas where a decrease of gray matter has been seen before surgery. Flipping the data of patients with left hip OA (and therefore normalizing for the side of the pain) had only little impact on the results but additionally showed a decrease of gray matter in the Heschl’s gyrus and Precuneus that we cannot easily explain and, as no a priori hypothesis exists, regard with great caution. However, the difference seen between patients and healthy controls at scan I was still observable in the cross-sectional analysis at scan IV. The relative increase of gray matter over time is therefore subtle, i.e. not sufficiently distinct to have an effect on the cross sectional analysis, a finding that has already been shown in studies investigating experience dependant plasticity [30], [31]. We note that the fact that we show some parts of brain-changes due to chronic pain to be reversible does not exclude that some other parts of these changes are irreversible.

Interestingly, we observed that the gray matter decrease in the ACC in chronic pain patients before surgery seems to continue 6 weeks after surgery (scan II) and only increases towards scan III and IV, possibly due to post-surgery pain, or decrease in motor function. This is in line with the behavioural data of the physical mobility score included in the NHP, which post-operatively did not show any significant change at time point II but significantly increased towards scan III and IV. Of note, our patients reported no pain in the hip after surgery, but experienced post-surgery pain in surrounding muscles and skin which was perceived very differently by patients. However, as patients still reported some pain at scan II, we also contrasted the first scan (pre-surgery) with scans III+IV (post-surgery), revealing an increase of gray matter in the frontal cortex and motor cortex. We note that this contrast is less stringent because of less scans per condition (pain vs. non-pain). When we lowered the threshold we repeat what we have found using contrast of I+II vs. III+IV.

Our data strongly suggest that gray matter alterations in chronic pain patients, which are usually found in areas involved in supraspinal nociceptive processing [4] are neither due to neuronal atrophy nor brain damage. The fact that these changes seen in the chronic pain state do not reverse completely could be explained with the relatively short period of observation (one year after operation versus a mean of seven years of chronic pain before the operation). Neuroplastic brain changes that may have developed over several years (as a consequence of constant nociceptive input) need probably more time to reverse completely. Another possibility why the increase of gray matter can only be detected in the longitudinal data but not in the cross-sectional data (i.e. between cohorts at time point IV) is that the number of patients (n = 20) is too small. It needs to be pointed out that the variance between brains of several individuals is quite large and that longitudinal data have the advantage that the variance is relatively small as the same brains are scanned several times. Consequently, subtle changes will only be detectable in longitudinal data [30], [31], [32]. Of course we cannot exclude that these changes are at least partly irreversible although that is unlikely, given the findings of exercise specific structural plasticity and reorganisation [4], [12], [30], [33], [34]. To answer this question, future studies need to investigate patients repeatedly over longer time frames, possibly years.

We note that we can only make limited conclusions regarding the dynamics of morphological brain changes over time. The reason is that when we designed this study in 2007 and scanned in 2008 and 2009, it was not known whether structural changes would occur at all and for reasons of feasibility we chose the scan dates and time frames as described here. One could argue that the gray matter changes in time, which we describe for the patient group, might have happened in the control group as well (time effect). However, any changes due to aging, if at all, would be expected to be a decrease in volume. Given our a priori hypothesis, based on 9 independent studies and cohorts showing decreases in gray matter in chronic pain patients [7], [8], [9], [15], [24], [25], [26], [27], [28], we focussed on regional increases over time and therefore believe our finding not to be a simple time effect. Of note, we cannot rule out that the gray matter decrease over time that we found in our patient group could be due to a time effect, as we have not scanned our control group in the same time frame. Given the findings, future studies should aim at more and shorter time intervals, given that exercise dependant morphometric brain changes may occur as fast as after 1 week [32], [33].

**Figure 3:**
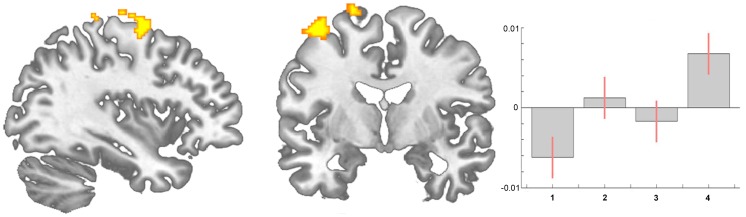
Statistical parametric maps demonstrating a significant increase of brain gray matter in motor areas (area 6) in patients with coxarthrosis before compared to after THR (longitudinal analysis, scan I<scan II<scan III<scan IV). Contrast estimates at x = 19, y = −12, z = 70.

In addition to the impact of the nociceptive aspect of pain on brain gray matter [17], [34] we observed that changes in motor function probably also contribute to the structural changes. We found motor and premotor areas (area 6) to increase over all time intervals (Figure 3). Intuitively this may be due to improvement of motor function over time as the patients were no more restricted in living a normal life. Notably we did not focus on motor function but an improvement in pain experience, given our original quest to investigate whether the well-known reduction in brain gray matter in chronic pain patients is in principle reversible. Consequently, we did not use specific instruments to investigate motor function. Nevertheless, (functional) motor cortex reorganization in patients with pain syndromes is well documented [35], [36], [37], [38]. Moreover, the motor cortex is one target in therapeutic approaches in medically intractable chronic pain patients using direct brain stimulation [39], [40], transcranial direct current stimulation [41], and repetitive transcranial magnetic stimulation [42], [43]. The exact mechanisms of such modulation (facilitation vs. inhibition, or simply interference in the pain-related networks) are not yet elucidated [40]. A recent study demonstrated that a specific motor experience can alter the structure of the brain [13]. Synaptogenesis, reorganisation of movement representations and angiogenesis in motor cortex may occur with special demands of a motor task. Tsao et al. showed reorganisation in the motor cortex of patients with chronic low back pain that seem to be back pain-specific [44] and Puri et al. observed a reduction in left supplemental motor area gray matter in fibromyalgia sufferers [45]. Our study was not designed to disentangle the different factors that may change the brain in chronic pain but we interpret our data concerning the gray matter changes that they do not exclusively mirror the consequences of constant nociceptive input. In fact, a recent study in neuropathic pain patients pointed out abnormalities in brain regions that encompass emotional, autonomic, and pain perception, implying that they play a critical role in the global clinical picture of chronic pain [28].

Two recent pilot studies focussed on hip replacement therapy in osteoarthritis patients, the only chronic pain syndrome which is principally curable with total hip replacement [17], [46] and these data are flanked by a very recent study in chronic low back pain patients [47]. These studies need to be seen in the light of several longitudinal studies investigating experience-dependent neuronal plasticity in humans on a structural level [30], [31] and a recent study on structural brain changes in healthy volunteers experiencing repeated painful stimulation [34]. The key message of all these studies is that the main difference in the brain structure between pain patients and controls may recede when the pain is cured. However, it must be taken into account that it is simply not clear whether the changes in chronic pain patients are solely due to nociceptive input or due to the consequences of pain or both. It is more than likely that behavioural changes, such as deprivation or enhancement of social contacts, agility, physical training and life style changes are sufficient to shape the brain [6], [12], [28], [48]. Particularly depression as a co-morbidity or consequence of pain is a key candidate to explain the differences between patients and controls. A small group of our patients with OA showed mild to moderate depressive symptoms that changed with time. We did not find the structural alterations to covary significantly with the BDI-score but the question arises how many other behavioural changes due to the absence of pain and motor improvement may contribute to the results and to what extent they do. These behavioural changes can possibly influence a gray matter decrease in chronic pain as well as a gray matter increase when pain is gone.

Another important factor which may bias our interpretation of the results is the fact that nearly all patients with chronic pain took medications against pain, which they stopped when they were pain free. One could argue that NSAIDs such as diclofenac or ibuprofen have some effects on neural systems and the same holds true for opioids, antiepileptics and antidepressants, medications which are frequently used in chronic pain therapy. The impact of pain killers and other medications on morphometric findings may well be important (48). No study so far has shown effects of pain medication on brain morphology but several papers found that changes in brain structure in chronic pain patients are neither solely explained by pain related inactivity [15], nor by pain medication [7], [9], [49]. However, specific studies are lacking. Further research should focus the experience-dependent changes in cortical plasticity, which may have vast clinical implications for the treatment of chronic pain.

We also found decreases of gray matter in the longitudinal analysis, possibly due to reorganisation processes that accompany changes in motor function and pain perception. There is little information available about longitudinal changes in brain gray matter in pain conditions, for this reason we have no hypothesis for a gray matter decrease in these areas after the operation. Teutsch et al. [25] found an increase of brain gray matter in the somatosensory and midcingulate cortex in healthy volunteers that experienced painful stimulation in a daily protocol for eight consecutive days. The finding of gray matter increase following experimental nociceptive input overlapped anatomically to some degree with the decrease of brain gray matter in this study in patients that were cured of long-lasting chronic pain. This implies that nociceptive input in healthy volunteers leads to exercise dependant structural changes, as it possibly does in patients with chronic pain, and that these changes reverse in healthy volunteers when nociceptive input stops. Consequently, the decrease of gray matter in these areas seen in patients with OA could be interpreted to follow the same fundamental process: exercise dependant changes brain changes [50]. As a non-invasive procedure, MR Morphometry is the ideal tool for the quest to find the morphological substrates of diseases, deepening our understanding of the relationship between brain structure and function, and even to monitor therapeutic interventions. One of the great challenges in the future is to adapt this powerful tool for multicentre and therapeutic trials of chronic pain.

### Limitations of this Study

Although this study is an extension of our previous study expanding the follow-up data to 12 months and investigating more patients, our principle finding that morphometric brain changes in chronic pain are reversible is rather subtle. The effect sizes are small (see above) and the effects are partly driven by a further reduction of regional brain gray matter volume at the time-point of scan 2. When we exclude the data from scan 2 (directly after the operation) only significant increases in brain gray matter for motor cortex and frontal cortex survive a threshold of p<0.001 uncorrected (Table 3).

**Table 3:** Longitudinal data, significant increase in gray matter comparing scan 1 with scan 3 and 4 (excluding scan 2) in patients with chronic pain due to primary hip osteoarthritis.

				MNI	
Anatomical locations	X	y	z	Peak t-scores	number of voxels
Right precentral gyrus	48	−6	62	3.61	419
Right precentral gyrus	24	−17	80	3.50	200
Right superior frontal gyrus, Area 6	17	0	74	4.00	98
Left superior medial gyrus	−9	46	24	3.93	65
Right postcentral gyrus	40	−32	71	3.59	3
Left cerebellum	−10	−63	−22	3.51	17
Left middle frontal gyrus	−34	9	64	3.42	8
Right precentral gyrus	26	−17	78	3.36	2

The changes are tabulated in terms of the brain region and the corresponding Brodmann’s area (BA). The x, y, z co-ordinates are according to the MNI atlas. Significance level p<0.001, uncorrected.

### Conclusion

It is not possible to distinguish to what extent the structural alterations we observed are due to changes in nociceptive input, changes in motor function or medication consumption or changes in well-being as such. Masking the group contrasts of the first and last scan with each other revealed much less differences than expected. Presumably, brain alterations due to chronic pain with all consequences are developing over quite a long time course and may also need some time to revert. Nevertheless, these results reveal processes of reorganisation, strongly suggesting that chronic nociceptive input and motor impairment in these patients leads to altered processing in cortical regions and consequently structural brain changes which are in principle reversible.

## References

[1] Woolf CJ, Salter MW (2000) Neuronal plasticity: increasing the gain in pain. Science 288: 1765–1769. 10846153 10.1126/science.288.5472.1765

[2] Flor H, Nikolajsen L, Staehelin Jensen T (2006) Phantom limb pain: a case of maladaptive CNS plasticity? Nat Rev Neurosci 7: 873–881. 17053811 10.1038/nrn1991

[3] Wrigley PJ, Gustin SM, Macey PM, Nash PG, Gandevia SC, et al (2009) Anatomical changes in human motor cortex and motor pathways following complete thoracic spinal cord injury. Cereb Cortex 19: 224–232. 18483004 10.1093/cercor/bhn072

[4] May A (2008) Chronic pain may change the structure of the brain. Pain 137: 7–15. 18410991 10.1016/j.pain.2008.02.034

[5] May A (2009) Morphing voxels: the hype around structural imaging of headache patients. Brain. 10.1093/brain/awp116 19443629

[6] Apkarian AV, Baliki MN, Geha PY (2009) Towards a theory of chronic pain. Prog Neurobiol 87: 81–97. 18952143 10.1016/j.pneurobio.2008.09.018 PMC2650821

[7] Apkarian AV, Sosa Y, Sonty S, Levy RM, Harden RN, et al (2004) Chronic back pain is associated with decreased prefrontal and thalamic gray matter density. J Neurosci 24: 10410–10415. 15548656 10.1523/JNEUROSCI.2541-04.2004 PMC6730296

[8] Rocca MA, Ceccarelli A, Falini A, Colombo B, Tortorella P, et al (2006) Brain gray matter changes in migraine patients with T2-visible lesions: a 3-T MRI study. Stroke 37: 1765–1770. 16728687 10.1161/01.STR.0000226589.00599.4d

[9] Kuchinad A, Schweinhardt P, Seminowicz DA, Wood PB, Chizh BA, et al (2007) Accelerated brain gray matter loss in fibromyalgia patients: premature aging of the brain? J Neurosci 27: 4004–4007. 17428976 10.1523/JNEUROSCI.0098-07.2007 PMC6672521

[10] Tracey I, Bushnell MC (2009) How neuroimaging studies have challenged us to rethink: is chronic pain a disease? J Pain 10: 1113–1120. 19878862 10.1016/j.jpain.2009.09.001

[11] Franke K, Ziegler G, Kloppel S, Gaser C (2010) Estimating the age of healthy subjects from T1-weighted MRI scans using kernel methods: exploring the influence of various parameters. Neuroimage 50: 883–892. 20070949 10.1016/j.neuroimage.2010.01.005

[12] Draganski B, May A (2008) Training-induced structural changes in the adult human brain. Behav Brain Res 192: 137–142. 18378330 10.1016/j.bbr.2008.02.015

[13] Adkins DL, Boychuk J, Remple MS, Kleim JA (2006) Motor training induces experience-specific patterns of plasticity across motor cortex and spinal cord. J Appl Physiol 101: 1776–1782. 16959909 10.1152/japplphysiol.00515.2006

[14] Duerden EG, Laverdure-Dupont D (2008) Practice makes cortex. J Neurosci 28: 8655–8657. 18753364 10.1523/JNEUROSCI.2650-08.2008 PMC6670828

[15] Draganski B, Moser T, Lummel N, Ganssbauer S, Bogdahn U, et al (2006) Decrease of thalamic gray matter following limb amputation. Neuroimage 31: 951–957. 16520065 10.1016/j.neuroimage.2006.01.018

[16] Nikolajsen L, Brandsborg B, Lucht U, Jensen TS, Kehlet H (2006) Chronic pain following total hip arthroplasty: a nationwide questionnaire study. Acta Anaesthesiol Scand 50: 495–500. 16548863 10.1111/j.1399-6576.2006.00976.x

[17] Rodriguez-Raecke R, Niemeier A, Ihle K, Ruether W, May A (2009) Brain gray matter decrease in chronic pain is the consequence and not the cause of pain. J Neurosci 29: 13746–13750. 19889986 10.1523/JNEUROSCI.3687-09.2009 PMC6666725

[18] Beck AT, Ward CH, Mendelson M, Mock J, Erbaugh J (1961) An inventory for measuring depression. Arch Gen Psychiatry 4: 561–571. 13688369 10.1001/archpsyc.1961.01710120031004

[19] Franke G (2002) Die Symptom-Checkliste nach L.R. Derogatis - Manual. Göttingen Beltz Test Verlag.

[20] Geissner E (1995) The Pain Perception Scale–a differentiated and change-sensitive scale for assessing chronic and acute pain. Rehabilitation (Stuttg) 34: XXXV–XLIII. 8570898

[21] Bullinger M, Kirchberger I (1998) SF-36 - Fragebogen zum Gesundheitszustand. Hand-anweisung. Göttingen: Hogrefe.

[22] Ashburner J, Friston KJ (2000) Voxel-based morphometry–the methods. Neuroimage 11: 805–821. 10860804 10.1006/nimg.2000.0582

[23] Good CD, Johnsrude IS, Ashburner J, Henson RN, Friston KJ, et al (2001) A voxel-based morphometric study of ageing in 465 normal adult human brains. Neuroimage 14: 21–36. 11525331 10.1006/nimg.2001.0786

[24] Baliki MN, Chialvo DR, Geha PY, Levy RM, Harden RN, et al (2006) Chronic pain and the emotional brain: specific brain activity associated with spontaneous fluctuations of intensity of chronic back pain. J Neurosci 26: 12165–12173. 17122041 10.1523/JNEUROSCI.3576-06.2006 PMC4177069

[25] Lutz J, Jager L, de Quervain D, Krauseneck T, Padberg F, et al (2008) White and gray matter abnormalities in the brain of patients with fibromyalgia: a diffusion-tensor and volumetric imaging study. Arthritis Rheum 58: 3960–3969. 19035484 10.1002/art.24070

[26] Wrigley PJ, Gustin SM, Macey PM, Nash PG, Gandevia SC, et al (2008) Anatomical Changes in Human Motor Cortex and Motor Pathways following Complete Thoracic Spinal Cord Injury. Cereb Cortex 19: 224–232. 18483004 10.1093/cercor/bhn072

[27] Schmidt-Wilcke T, Hierlmeier S, Leinisch E (2010) Altered Regional Brain Morphology in Patients With Chronic Facial Pain. Headache. 10.1111/j.1526-4610.2010.01637.x 20236343

[28] Geha PY, Baliki MN, Harden RN, Bauer WR, Parrish TB, et al (2008) The brain in chronic CRPS pain: abnormal gray-white matter interactions in emotional and autonomic regions. Neuron 60: 570–581. 19038215 10.1016/j.neuron.2008.08.022 PMC2637446

[29] Brazier J, Roberts J, Deverill M (2002) The estimation of a preference-based measure of health from the SF-36. J Health Econ 21: 271–292. 11939242 10.1016/s0167-6296(01)00130-8

[30] Draganski B, Gaser C, Busch V, Schuierer G, Bogdahn U, et al (2004) Neuroplasticity: changes in grey matter induced by training. Nature 427: 311–312. 14737157 10.1038/427311a

[31] Boyke J, Driemeyer J, Gaser C, Buchel C, May A (2008) Training-induced brain structure changes in the elderly. J Neurosci 28: 7031–7035. 18614670 10.1523/JNEUROSCI.0742-08.2008 PMC6670504

[32] Driemeyer J, Boyke J, Gaser C, Buchel C, May A (2008) Changes in gray matter induced by learning–revisited. PLoS ONE 3: e2669. 18648501 10.1371/journal.pone.0002669 PMC2447176

[33] May A, Hajak G, Ganssbauer S, Steffens T, Langguth B, et al (2007) Structural brain alterations following 5 days of intervention: dynamic aspects of neuroplasticity. Cereb Cortex 17: 205–210. 16481564 10.1093/cercor/bhj138

[34] Teutsch S, Herken W, Bingel U, Schoell E, May A (2008) Changes in brain gray matter due to repetitive painful stimulation. Neuroimage 42: 845–849. 18582579 10.1016/j.neuroimage.2008.05.044

[35] Flor H, Braun C, Elbert T, Birbaumer N (1997) Extensive reorganization of primary somatosensory cortex in chronic back pain patients. Neurosci Lett 224: 5–8. 9132689 10.1016/s0304-3940(97)13441-3

[36] Flor H, Denke C, Schaefer M, Grusser S (2001) Effect of sensory discrimination training on cortical reorganisation and phantom limb pain. Lancet 357: 1763–1764. 11403816 10.1016/S0140-6736(00)04890-X

[37] Swart CM, Stins JF, Beek PJ (2009) Cortical changes in complex regional pain syndrome (CRPS). Eur J Pain 13: 902–907. 19101181 10.1016/j.ejpain.2008.11.010

[38] Maihofner C, Baron R, DeCol R, Binder A, Birklein F, et al (2007) The motor system shows adaptive changes in complex regional pain syndrome. Brain 130: 2671–2687. 17575278 10.1093/brain/awm131

[39] Fontaine D, Hamani C, Lozano A (2009) Efficacy and safety of motor cortex stimulation for chronic neuropathic pain: critical review of the literature. J Neurosurg 110: 251–256. 18991496 10.3171/2008.6.17602

[40] Levy R, Deer TR, Henderson J (2010) Intracranial neurostimulation for pain control: a review. Pain Physician 13: 157–165. 20309382

[41] Antal A, Brepohl N, Poreisz C, Boros K, Csifcsak G, et al (2008) Transcranial direct current stimulation over somatosensory cortex decreases experimentally induced acute pain perception. Clin J Pain 24: 56–63. 18180638 10.1097/AJP.0b013e318157233b

[42] Teepker M, Hotzel J, Timmesfeld N, Reis J, Mylius V, et al (2010) Low-frequency rTMS of the vertex in the prophylactic treatment of migraine. Cephalalgia 30: 137–144. 19515124 10.1111/j.1468-2982.2009.01911.x

[43] O’Connell N, Wand B, Marston L, Spencer S, Desouza L (2010) Non-invasive brain stimulation techniques for chronic pain. A report of a Cochrane systematic review and meta-analysis. Eur J Phys Rehabil Med 47: 309–326. 21494222

[44] Tsao H, Galea MP, Hodges PW (2008) Reorganization of the motor cortex is associated with postural control deficits in recurrent low back pain. Brain 131: 2161–2171. 18669505 10.1093/brain/awn154

[45] Puri BK, Agour M, Gunatilake KD, Fernando KA, Gurusinghe AI, et al (2010) Reduction in left supplementary motor area grey matter in adult female fibromyalgia sufferers with marked fatigue and without affective disorder: a pilot controlled 3-T magnetic resonance imaging voxel-based morphometry study. J Int Med Res 38: 1468–1472. 20926020 10.1177/147323001003800429

[46] Gwilym SE, Fillipini N, Douaud G, Carr AJ, Tracey I (2010) Thalamic atrophy associated with painful osteoarthritis of the hip is reversible after arthroplasty; a longitudinal voxel-based-morphometric study. Arthritis Rheum. 10.1002/art.27585 20518076

[47] Seminowicz DA, Wideman TH, Naso L, Hatami-Khoroushahi Z, Fallatah S, et al (2011) Effective treatment of chronic low back pain in humans reverses abnormal brain anatomy and function. J Neurosci 31: 7540–7550. 21593339 10.1523/JNEUROSCI.5280-10.2011 PMC6622603

[48] May A, Gaser C (2006) Magnetic resonance-based morphometry: a window into structural plasticity of the brain. Curr Opin Neurol 19: 407–411. 16914981 10.1097/01.wco.0000236622.91495.21

[49] Schmidt-Wilcke T, Leinisch E, Straube A, Kampfe N, Draganski B, et al (2005) Gray matter decrease in patients with chronic tension type headache. Neurology 65: 1483–1486. 16275843 10.1212/01.wnl.0000183067.94400.80

[50] May A (2009) Morphing voxels: the hype around structural imaging of headache patients. Brain 132(Pt 6): 1419–1425. 10.1093/brain/awp116 19443629

